# Strategy of optical path of daylight signal into tissues in cold-season turfgrasses using small, concave silica bodies

**DOI:** 10.1038/s41598-018-28159-6

**Published:** 2018-07-06

**Authors:** Shigeru Yamanaka, Hisanao Usami, Keiko Kakegawa, Satoshi Yoneda, Kenichi Fukuda, Katsumi Yoshino, Nobuaki Hayashida, Yasushi Murakami, Hideaki Morikawa

**Affiliations:** 1 Faculty of Textile Science and Technology, Shinshu University, 3-15-1 Tokida, Ueda, Nagano 386-8567 Japan; 2 Research Center for Supports to Advanced Science, Ueda Branch of Division of Instrumental Research, Shinshu University, 3-15-1 Tokida, Ueda, Nagano 386-8567 Japan; 3 Shimane Institute for Industrial Technology, 1 Hokuryo-cho, Matsue, Shimane 690-0816 Japan

## Abstract

Plants incorporate inorganic materials (biominerals), such as silica, into their various components. Plants belonging to the order *Poales*, like rice plants and turfgrasses, show comparatively high rates of silicon accumulation, mainly in the form of silica bodies. This work aims to determine the shapes and roles of these silica bodies by microscopic observation and optical simulation. We have previously found convex silica bodies on the leaves of rice plants and hot-season turfgrasses (adapted to hot-seasons). These silica bodies enabled light reflection and ensured reduction of the photonic density of states, which presumably prevented the leaves from overheating, as suggested by theoretical optical analyses. The silica bodies have been considered to have the functions of reinforcement of the plant body. The present work deals with cold-season turfgrasses, which were found to have markedly different silica bodies, cuboids with a concave top surface. They presumably acted as small windows for introducing light into the tissues, including the vascular bundles in the leaves. The area of the silica bodies was calculated to be about 5% of the total surface area of epidermis, which limits the thermal radiation of the silica bodies. We found that the light signal introduced through the silica bodies diffused in the organs even reaching the vascular bundles, the physiological functions of this phenomena remain as future problems. Light signal in this case is not related with energy which heat the plant but sensing outer circumstances to respond to them.

## Introduction

Living organisms utilize inorganic materials (biominerals), such as silicon and calcium, in their components. Silicon, which is ubiquitous in soil, is taken up by plants in the form of various soluble compounds, such as S_i_(OH)_4_.Silica bodies of *Oryza*^1–3^ and *Equisetaceae*^4–6^ have been studied to determine where in the plant they occur and their physiological function.

It has been found that silicon play a role in providing protection against predation^7,8^. Silicon may interact with several key components of plant stresses signaling systems ultimately leading to induced resistance against pathogenic fungi^7^ and S_i_-mediated protection involves mechanism other than salicylic acid-dependent defense responses against pathogenic fungi mike mildew^8^.

Silica also imparts useful optical properties to plants. Several communications have been published on the optical properties of plant silica in relation to plant functions. Agarie *et al*. reported that silica has no effect on the optical properties of rice leaves^9^, but we found concave form of silica bodies, which were confirmed by optical experiments to introduce light into the organs in diffused form. We also have reported analyses of silica bodies of plants belonging to the order *Poales*, like the rice plant and turfgrasses.

On the leaves of the rice plant are found convex silica bodies^1^, whose function is to impart certain optical properties to the plant, such as reflection of light and reduction of the photonic density of states. These properties presumably prevent the leaves of the rice plant from being heated above ∼20 °C^1^. A similar study we conducted on silica bodies of turfgrasses (hot-season type) revealed them to be convex and it was presumed from the results of silica bodies analyses of rice plant^1^ that they reflected light to cool the plant^10^. Silica structures deposited in epidermal cells of plant leaves were reported to reduce the heat load of the leaves^11^.

Trichomes with silica particles were found on the leaves of *Aphananthe aspera*, which appeared to help incident far-infrared light to propagate efficiently inside the trichomes and leaves. It is believed that this, in turn, helped to heat the plant^12^.

Silica bodies in rice plants have been reported to act as both a solar diffuser and a window: Silica plates were suggested to diffuse visible light effectively in the leaf blade, and fan-shaped silicas play a role in guiding light to chloroplasts^3^.

Turfgrasses can broadly be divided into two types: hot-season and cold-season turfgrasses. We have found that the silica microstructure of a leaf of a cold-season turfgrass, “Kentucky bluegrass” is quite different from that of a leaf of a hot-season turfgrass, “Tifton419”^10^ in which the warm-season turfgrass was found to have characteristic silica bodies which were speculated to reflect light to cool down the plants from the photo of silica bodies which were found to arranged in roughly regular mode and cool-season turfgrass were found to have characteristically scrobiculate micro-structure in which air may be retained, and these pockets might insulate the plant from low temperature. At this stage, however, silica bodies mentioned in this paper were not found.

**Figure 1:**
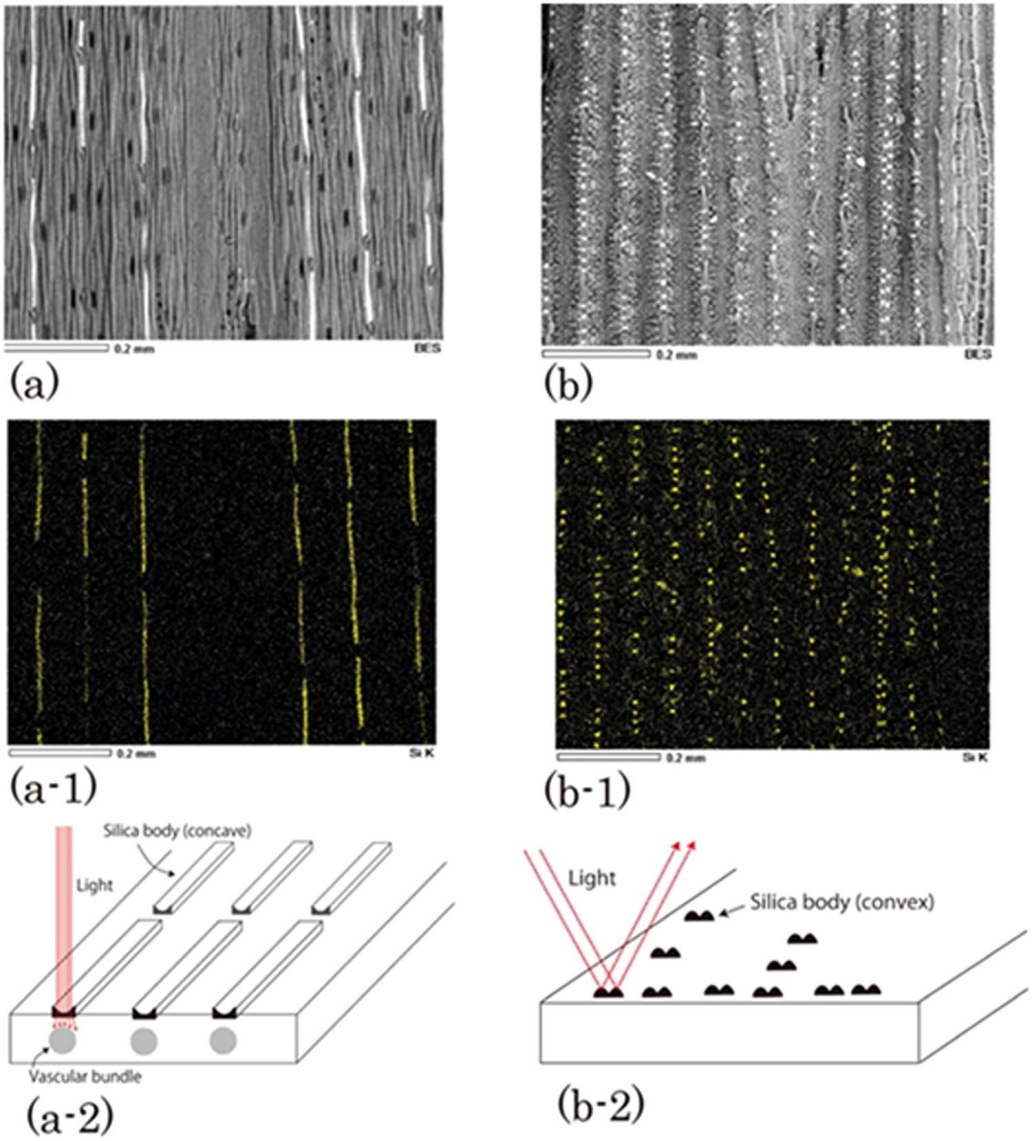
Epidermal surface of leaves of cold- and hot-season turfgrasses. (**a**) Scanning electron microscopy (SEM) images of the surface of a Kentucky turfgrass leaf, with silica bodies shown as white bars in cold-season plants. (a-1) Energy-dispersive X-ray spectroscopy (EDS) analyses confirmed that these elongated bars were composed of silica. (a-2) Schematics of how light penetrates a leaf through the silica cuboids with a concave top in cold-season turfgrass. (**b**) SEM image of the surface of a Korai leaf, with silica bodies shown as white dots in this hot-season plant. An enlarged SEM image is shown in Fig. S3. (b-1) EDS analyses confirmed that these dots were composed of silica. (b-2) Schematics of how light reflects a leaf through the convex silica in hot-season turfgrass.

The microstructure of silica for cold-season is unknown, while for hot season turfgrass the microstructure of convex silica is effective for reflecting the light as shown in Fig. 1(b2). Our goal was to characterize the properties of the silica bodies of cold-season turfgrasses.

## Results

### Microscopy of turfgrass leaves

We studied leaves of turfgrasses using microscopy and discovered that their silica bodies had a cuboid shape, with a concave-top surface, in contrast to the convex silica bodies of hot-season turfgrasses like leaf of Korai. Careful analysis of the present silica bodies revealed their important optical functions.

We developed a system for passing light through silica bodies to determine their functions. The silica bodies of cold-season turfgrasses were examined by using the leaves of Kentucky bluegrass (*Poales pratensis*) and perennial turfgrass (*Lollium perenne*) along with those of the hot-season turfgrass Korai (*Zoysia pacifica*). The epidermal structures of the leaves were examined microscopically to detect the presence of silica bodies (Figs 1 and S1). The morphologies of the silica bodies of the leaves of the cold-season turfgrasses Kentucky blue grass and perennial rye grass were found to be fundamentally similar.

**Figure 2:**
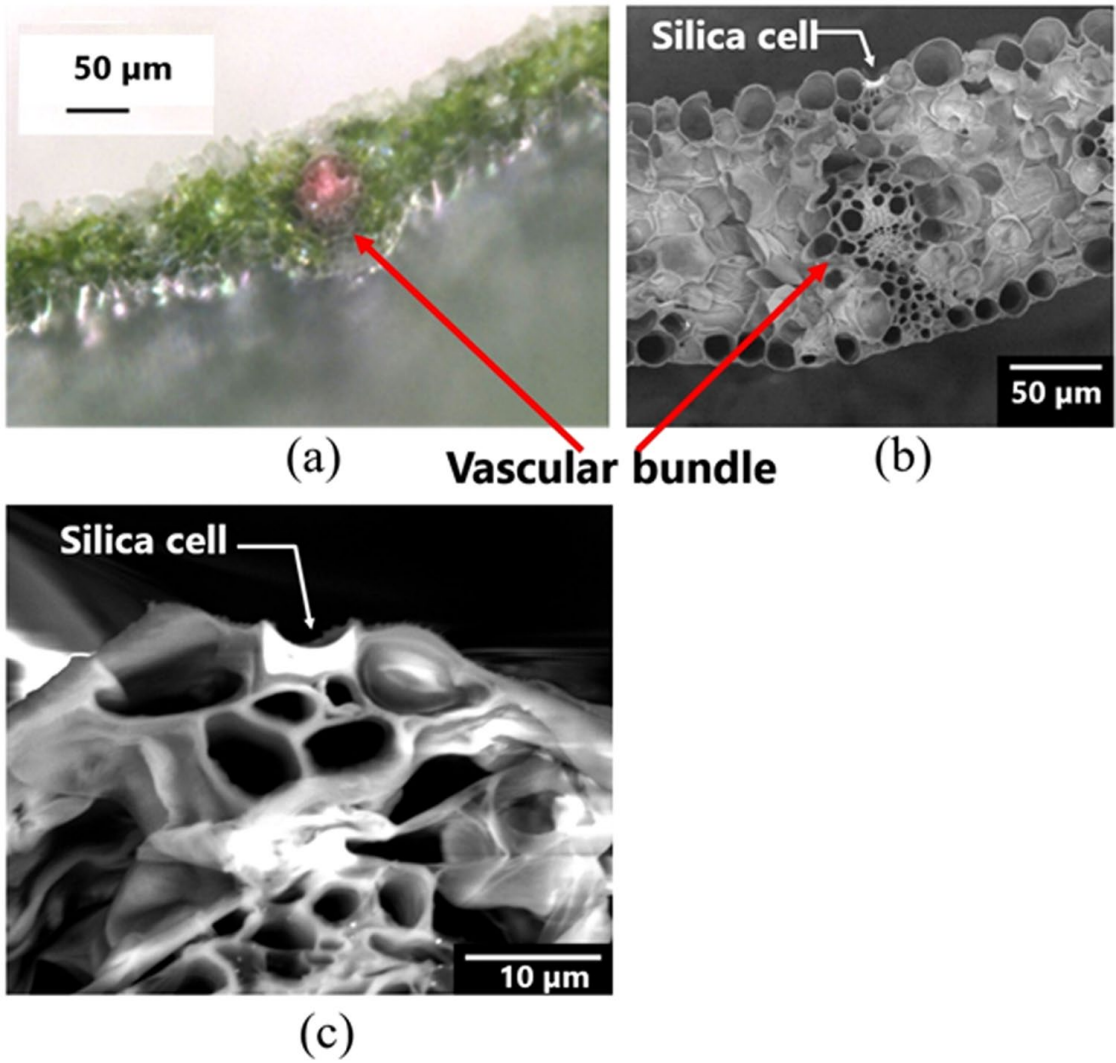
Micrograph of the cross-section of a cold-season turfgrass leaf. (**a**–**c**) Kentucky turfgrass. (**a**) A vascular bundle revealed through optical microscopy by the pink color associated with eosine. Green zones are chlorophyll. (**b**) SEM image of a similar portion of the leaf. (**c**) SEM image of a section of a leaf containing a silica body. The width of the concave body is about 8 µm. This was selected as a typical silica cell (body) sliced in a way of least artefact. Figure S4 (**b**) shows the photo of the sliced body. Figure S5 indicates the dimensions of (**b**) and others.

A Kentucky turfgrass leaf specimen was sliced to view the interior structure of the leaf (Fig. 2).

Cuboids with a concave top surface are visible in images (Fig. 1(a-1)) of the cold-season plant leaves. However, their function remains unclear. We examined the microstructure of Kentucky and perennial turfgrass leaves. They were found to have a similar microstructure (Fig. S1).

### Optical analysis of leaves having silica cell

The small surface area of the silica cell indicates that the plants harvest light signal so efficiently that only a very small area of about 5% of the total area, as determined using software “ImageJ” (National Institute of Health) as shown in Figs S6 and S7. Moreover, the energy inside the organ maybe lost at lower level to prevent the plant from cooling. In the optical experiments, we irradiated the interior of a Kentucky turfgrass leaf through a concave silica cells using an optical microfiber to investigate how light penetrates the leaf. For this purpose, we created a micro-miniature imaging optical lens by melting an extremely fine glass fiber end^13,14^. The iameter of the optical microfiber was approximately 50 µm. The diameter of the light beam was 20 µm, and we created an optical fiber having a width as small as 3 µm, less than the width of a concave cells of the turfgrass. (We aimed the light so that most of it would penetrate the leaves). We hypothesized that the silica cells in a concave lens form function as windows, introducing sunlight into the plant’s organs, e.g., its vascular bundles. Thus, we investigated how artificial light would pass through the concave cells and how it would irradiate the inner cells of the leaf, including those in the vascular bundles located just beneath the cells (Fig. 2(a,b)). The position of the vascular bundles, through which water is absorbed from the roots, was determined by staining with pink eosine solution, as shown in Fig. 2(a). It seems that mesophylls containing chlorophyll are scarce in the space beneath the silica bodies.

**Figure 3:**
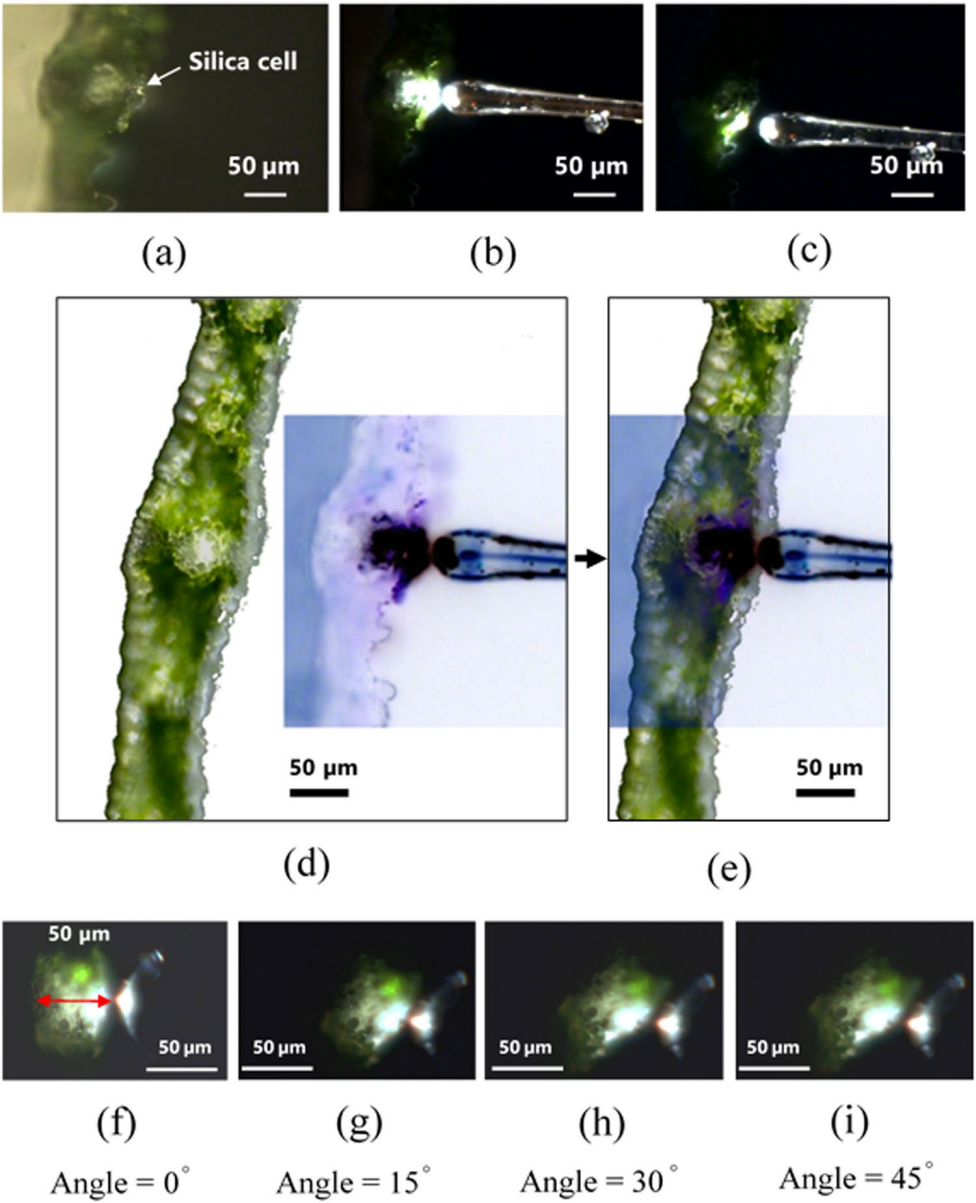
Photographs demonstrating the light path from the optical microfiber through the concave cell. (**a**) Details of the sliced leaf containing silica concave cell. (**b**) Detail of light irradiation experiments using an optical fiber placed on a silica cell. This photograph shows light passing at an angle of 0° (perpendicular to the leaf surface). (**c**) Similar photograph of an optical fiber placed 50 μm shifted along the vascular and the silica cell (control). The light irradiated a smaller area than the light irradiating the silica cell. (**d**,**e**) Confirmation of the positional relationship between the irradiated light and the vascular bundle in the cross-section of a turfgrass leaf. (**f**–**i**) Irradiation angles of 0, 15, 30, and 45°, respectively.

The position of irradiation of the plant with the optical fiber was varied from 0 to 45° in order to mimic the sun’s movement (clockwise). Likewise, the fiber was positioned and moved in a counterclockwise manner (0, 15, 30, and 45°), which yielded a similar pattern (data not shown). The results of irradiation tests using the light beam from an optical fiber are shown in Figs 3 and S2.

The results of irradiation tests using the light beam from an optical fiber are shown in Figs 3 and S2. The position of irradiation of the plant with the optical fiber was varied from 0 to 45° in order to mimic the sun’s movement (clockwise). Likewise, the fiber was positioned and moved in a counterclockwise manner (0, 15, 30, and 45°), which yielded a similar pattern (data not shown).

As shown in Fig. 3, light penetrated the cells and irradiated the interior of the organs in a scattered mode, reaching the chlorophyll-containing cells and the vascular bundles. Thus, our data suggest that sunlight passing through the cells should reach the surrounding plant tissues like mesophyll (which contains chlorophyll) and the vascular bundles. Generally, Plants time their flowering according to the availability of light. In the case of *Arabidopsis*, the photoreceptors present in the plant’s tissues were found to utilize light. It could be speculated that turfgrasses similarly would respond to light introduced through their silica cells, although further experimental evidence is necessary.

Ray tracing was simulated as below.

**Figure 4:**
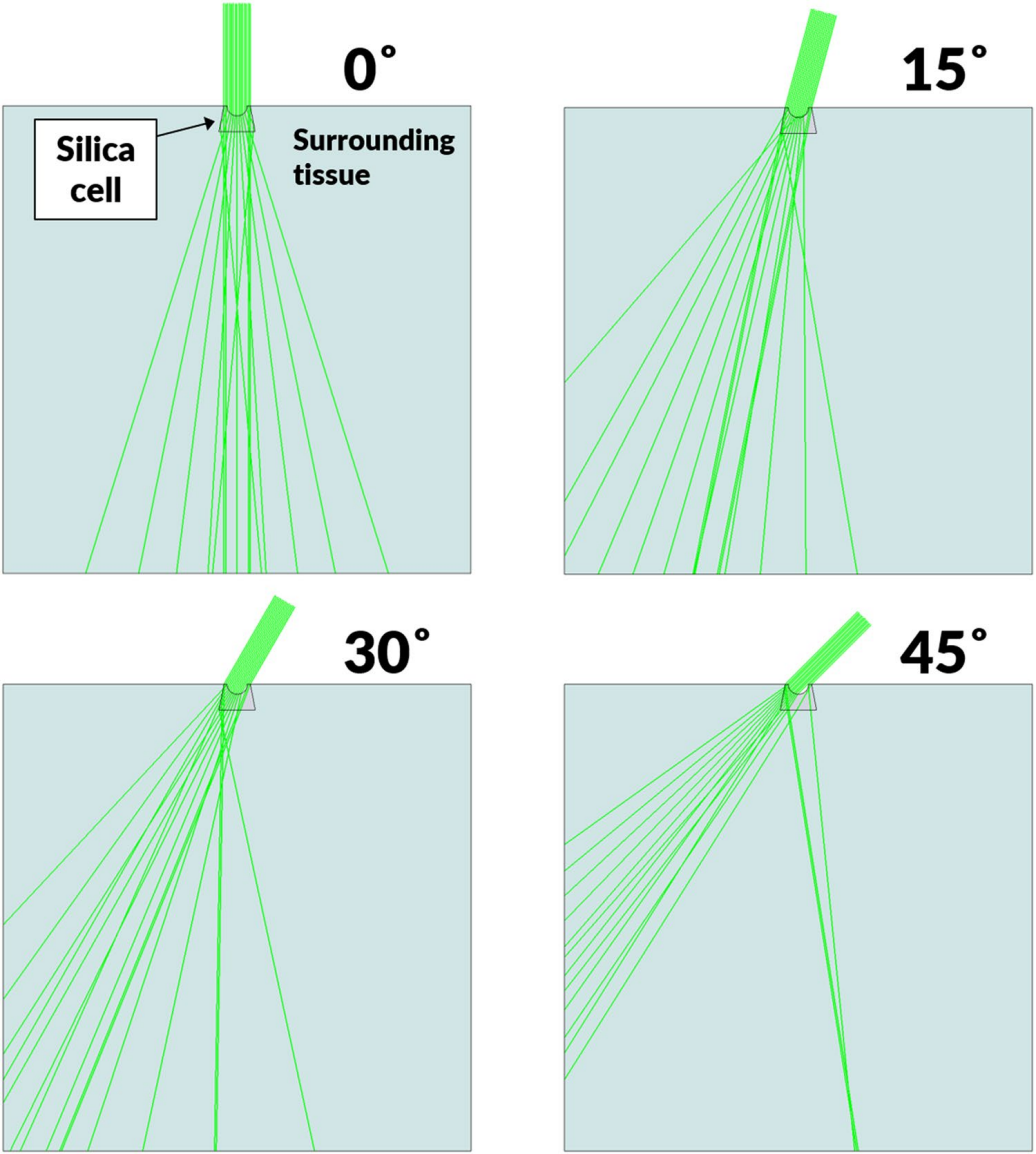
Results of ray tracing simulations, as calculated according to E. Hecht. *Optics*. International edition, Addison-Wesley, San Francisco 4th Edition (2002)^16^.

Figure 4 illustrates the model structure of the leaf surface of a cold-season turfgrass embedded with a concave cells. Ray tracing simulations were carried out, in which the light beam was focused from the top onto the upper surface of the lens at various incident angles. The irradiated area consisted of a silica cells with the dimensions shown in Fig. 3(d), along with the surrounding material and air. The refractive index of the silica cells was set to 1.427 (that of plant silica)^15^ and that of the surrounding material (which was assumed to be water) was set to 1.33. The behavior of the light rays at the interface was determined by Snell’s law.

The light path in the experiments may differ from that in the simulations, which assumed no texture of cell assembly. Figure 4 shows the results of the simulations, in which collimated light was introduced into homogeneous water. For simplicity, it was assumed that the cells underneath the convex lens had no texture. The experiments were performed by using optical fibers that emitted mixtures of collimated and refracted light into the multicellular leaf through the silica bodies.

## Discussion

Turfgrasses must preserve their heat during the cold-season. Experiments have revealed the light introduced through silica cell to be weak and scattered but capable of reaching even the vascular bundles. Morphologically, it was found that chlorophyll-containing mesophyll were scarce in the space beneath the silica cells, and this morphology may be facilitating the passage of light.

During our investigation of cold-season turfgrass of the order *Poales*, we discovered a new type of silica cell on the leaf surface. It is believed that this particular arrangement of silica cells render the plant well-adapted to cold climates.

It was demonstrated that in irradiation through a silica cells, 20-μm and 30-μm optical beams (concave silica cell width: 8 μm) yielded similar patterns, regardless of whether the beam diameter was larger or smaller than the concave cell diameter.

The silica cells were cuboid with a concave top surface. A plant having such silica cells was optically analyzed. The light path in these experiments could differ from that in the simulation, which assumed no textures of cell assembly.

The simulations are shown in Fig. 4, which assumed that collimated light was introduced into homogeneous water. The experiments were performed by using optical fibers that emitted mixtures of collimated and refracted light into a multicellular leaf through a silica cell. The discrepancy between the experimental results and simulation results may be due to the fact that while the light beams in the simulation are collimated, those from the optical fiber are a combination of collimated and refracted beams, leading to more light scattering in the case of the optical fiber, as shown in Fig. 3.

We found that the light signal introduced through the silica bodies diffused in the organs even reaching the vascular bundles. The physiological functions of this phenomena remain as future problems to be solved.

## Methods

### Plant resources

The turfgrasses (supplied by International Golf Management Co., Ltd. [Ibaraki, Japan]) used in this study included Kentucky, perennial (a cold-season type), and Korai (a hot-season type).

### Microscopic observation

AVHX digital microscope (Keyence Corp.) was used. Scanning electron microscopy (SEM). A JEOL JSM-6010LA. SEM equipped with an energy-dispersive spectroscopy (EDS) instrument was used.

### Observation of vascular bundles

The roots of the plants were immersed in an aqueous eosin Y solution (1 mM aqueous solution). Leaves that absorbed the pink-colored eosin were sliced and observed by an optical microscope.

### Irradiation of interior of turfgrass leaves using an optical fiber

Light was introduced into the leaves through the concave cells in the epidermis using an optical fiber with a diameter of ∼50 µm. The widths of the light beams used were approximately 20 µm. Light from the optical fiber was introduced perpendicular to the lens (0°) and at angles of 15, 30, 45, 60, 75, and 90° to the cells to mimic the natural movement of the sun. The light path was photographed for a sliced leaf sample.

## Electronic supplementary material

Supplementary Materials
