# Supplementary material for: Strategy of optical path of daylight signal into tissues in cold-season turfgrasses using small, concave silica bodies
Source: Sci Rep. 2018 Jul 6;8:10260. doi: 10.1038/s41598-018-28159-6 (PMC6035188; doi:10.1038/s41598-018-28159-6)
Supplement: Supplementary file 1 — Supplementary Materials [file 41598_2018_28159_MOESM1_ESM.pdf]

Strategy of optical path of daylight signal into tissues in cold-season turfgrasses using small, concave silica bodies

Shigeru Yamanaka[1*], Hisanao Usami[1], Keiko Kakegawa[2], Satoshi Yoneda[1], Kenichi Fukuda[3], Katsumi Yoshino[3], Nobuaki Hayashida[1], Yasushi Murakami[1], Hideaki Morikawa[1]

Supplementary Materials

1. Morphologies and optical experiments of silica cells

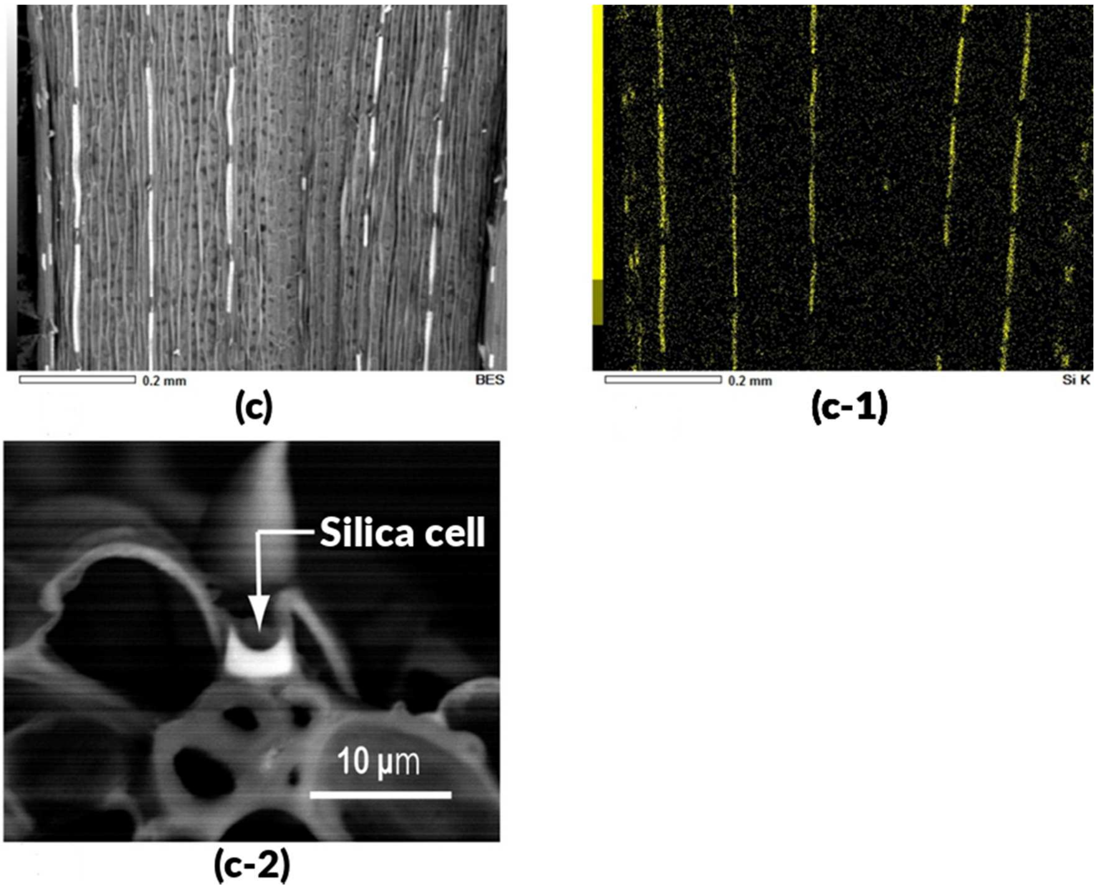

Fig. S1. Epidermal surface of leaves of cold-season perennial turfgrass.
(c) Scanning electron microscopy (SEM) images of the surface of perennial turfgrass leaves, with silica cells shown as white bars.
(c-1) Energy-dispersive X-ray spectroscopy (EDS) analysis confirmed that the elongated bars were composed of silica.
(c-2) SEM image of a leaf section containing a silica cells.

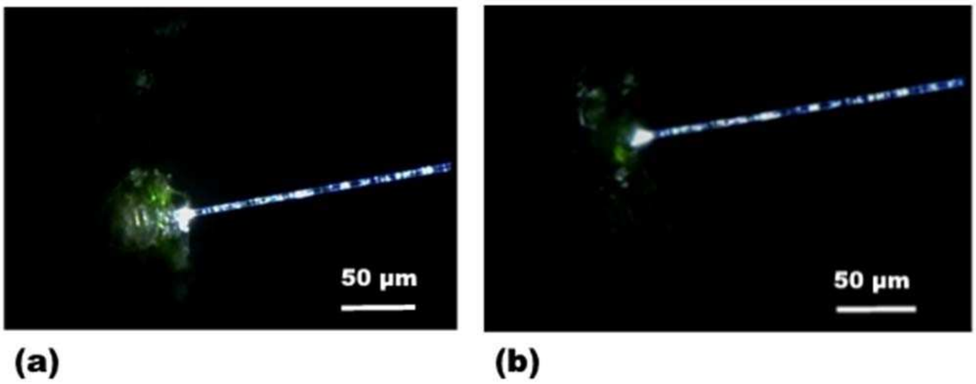

Fig. S2. Photographs showing the light path from an optical microfiber through the concave cells. The beam width was 3 μm. (a) Details of light irradiation experiments using an optical fiber placed on a cell. (b) Similar photograph of an optical fiber placed 50 μm from the periphery of the lens (control). The light irradiated a smaller area than the light that irradiated the silica cells.

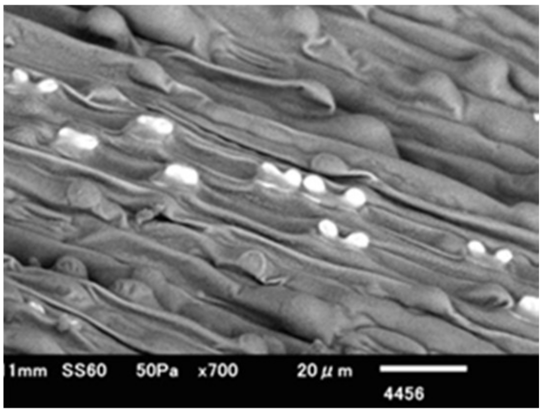

Fig. S3. SEM image of the leaf surface of the hot-season turfgrass Korai. The observed silica cells were confirmed to contain silicon by EDS analysis. They are convex, in contrast to the concave cold-season turfgrass silica cells. These structures resemble the structures found in rice plants.[1]

2. Analysis procedure for taking cross-sectional shapes of silica cells

1. We got SEM images for five cross-sectional part of Kentucky bluegrass samples (Fig. S4: (a)-(e)).
2. We converted these images to binary images (Fig. S5 (a)-(e)) in order to get the shape of silica cell.
3. We measured the shape of silica cells by using “ImageJ” which is an image analyzer developed by National Institute of Health (NIH), US*. * ImageJ: https://imagej.nih.gov/ij/
4. We constructed the cells dimensions in our simulation by referring to sample (b) as typical shape of them.

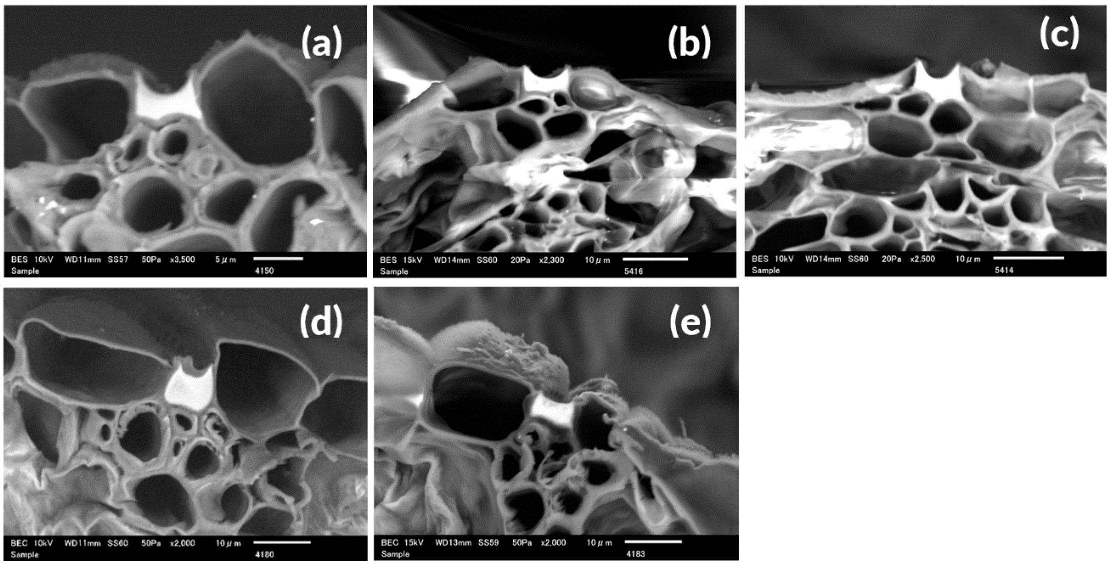

Fig. S4. SEM images for cross-sectional part of Kentucky bluegrass samples.

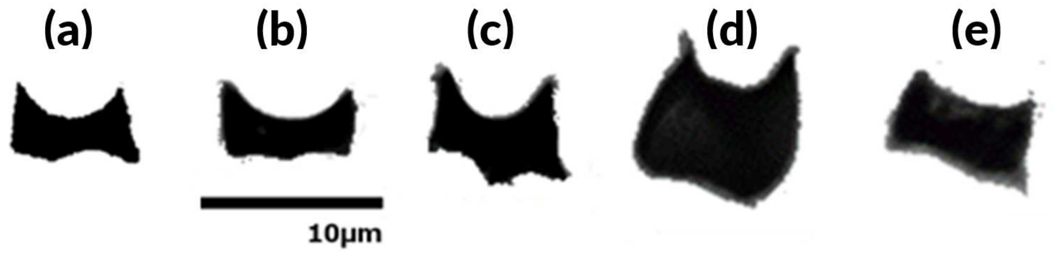

Fig. S5. Silica cell parts extracted from SEM images by using “ImageJ”.

3. Analysis procedure for ratio of Si area in Kentucky bluegrass surface

For simplicity, Kentucky bluegrass was selected for area calculation.

1. We got a SEM photo Fig. S6 (a) and EDX image Fig. S6 (b) of Kentucky bluegrass surface, firstly.
2. We cut area (Fig. S7 (a)) considering the fundamental unit in Si pattern from Fig. S6 (b).
3. We converted the EDX image Fig. S7 (a) to a binary image Fig. S7 (b) in order to separate the Si area (black part) and other area (withe part) clearly.
4. We calculated the ratio of Si area (Black part) by using “ImageJ” which is an image analyzer developed by National Institute of Health (NIH), US*.   * ImageJ: https://imagej.nih.gov/ij/
5. We got the ratio 5.0% by the ImageJ.

Analysis and calculation results in Fig. S7 (b)

Image size: 193*197=38,021 pixels
Total area of Black part (Si part): 1,905 pixels
Ratio of Black part: 1,905/38,021=5.0%

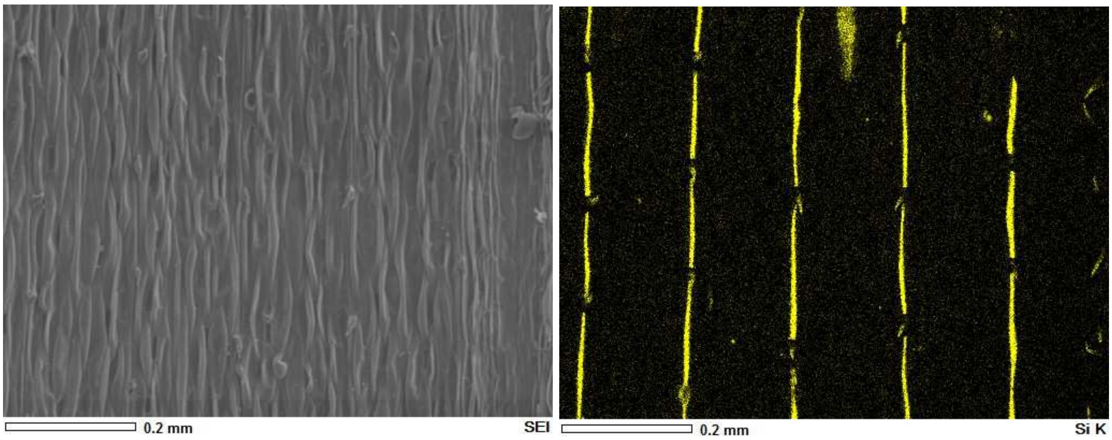

(a) SEM image (b) EDX image of Si

Fig. S6. Images of Kentucky blue grass surface (same area).

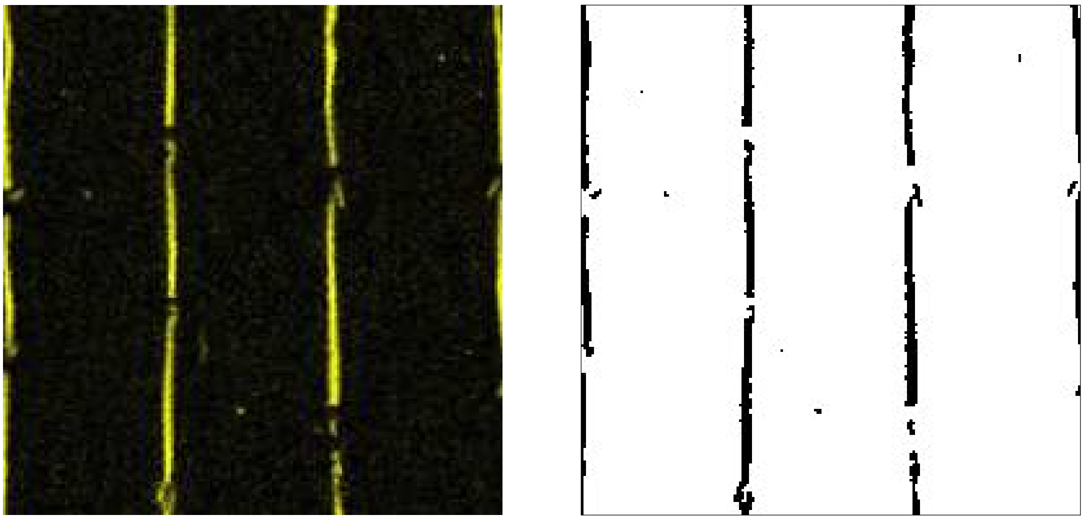

(a) Basic pattern cut from Fig. S6 (b) Binary image (Black area: Si)

Fig. S7. Image processing of a basic pattern of Si part.

4. Three-dimensional photo for surface and cross-section of Kentucky bluegrass and distributed part of silica body

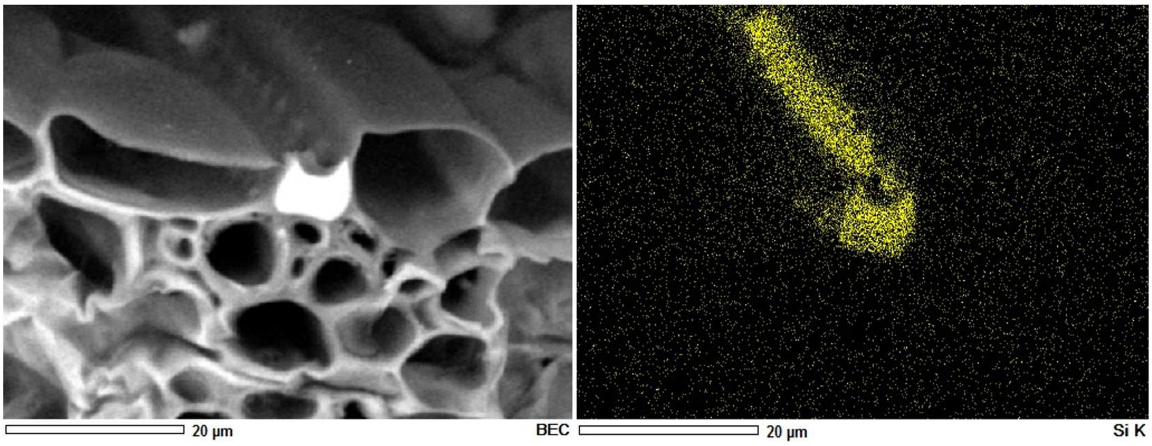

Fig. S8. Three-dimensional photo for silica body of specimen (6) in Fig. S4 and S5 (left; 3D image and right; EDX profile).
